# Serum IL-23 levels reflect a myeloid inflammatory signature and predict the response to apremilast in patients with psoriatic arthritis

**DOI:** 10.3389/fimmu.2024.1455134

**Published:** 2024-12-04

**Authors:** Maria De Santis, Antonio Tonutti, Natasa Isailovic, Francesca Motta, Radu Marian Rivara, Rita Ragusa, Giacomo M. Guidelli, Marta Caprioli, Angela Ceribelli, Daniela Renna, Nicoletta Luciano, Carlo Selmi

**Affiliations:** ^1^ Department of Biomedical Sciences, Humanitas University, Pieve Emanuele, Italy; ^2^ Rheumatology and Clinical Immunology, IRCCS Humanitas Research Hospital, Rozzano, Italy

**Keywords:** spondyloarthritis (including psoriatic arthritis), precision medicine, immunology, cytokines, innate lymphocyte, macrophages

## Abstract

**Background:**

The phosphodiesterase 4 (PDE4) inhibitor apremilast downregulates the production of IL-23 and other pro-inflammatory cytokines involved in the pathogenesis of psoriatic arthritis (PsA).

**Aim:**

To investigate the effects of apremilast on the production of cytokines by peripheral blood monocyte-derived macrophages, innate-like lymphocyte cells (ILCs), mucosal-associated invariant T (MAIT) cells, γδ T cells, natural killer (NK) cells, and NKT-like cells from patients with PsA manifesting different clinical responses to the treatment.

**Methods:**

Peripheral blood samples were obtained from patients with PsA at baseline and after 1 and 4 months of apremilast therapy (n = 23) and 20 controls with osteoarthritis. Cytokine expression in peripheral blood monocyte-derived macrophages and ILCs/MAIT/γδT/NK/NKT-like cells was tested by RT-PCR and FACS analyses, respectively; cytokine levels in culture supernatants and sera were analyzed by ELISA.

**Results:**

PsA monocyte-derived macrophages exhibited higher expressions of IL-23, IL-1β, and TNF-α, compared with OA controls, more profoundly in patients responding to apremilast. There were 17/23 (74%) PsA patients who were classified as responders to apremilast at 4 months, and a baseline serum IL-23 >1.4 pg/mL was associated with the responder status (AUC_ROC_ 0.79; sensitivity 100%, specificity 68%). Of note, apremilast led to a significantly reduced expression of IL-23 in peripheral blood monocyte-derived macrophages; IL-17 in ILC1 and in T cells of responder patients; IFN-γ in γδ T lymphocytes.

**Conclusion:**

An enhanced myeloid inflammatory signature characterizes PsA monocyte-derived macrophages, and serum IL-23 levels represent candidate biomarkers for PsA response to apremilast.

## Introduction

Psoriatic arthritis (PsA) is a chronic inflammatory disease with a heterogeneous clinical presentation encompassing different disease phenotypes, including arthritis, enthesitis, dactylitis, and axial involvement, often following skin and nail psoriasis (1). The pathogenesis of PsA is sustained by the interaction of both innate and adaptive immune mechanisms, involving innate cell subsets such as monocytes–macrophages, γδ T cells, natural killer (NK) cells, invariant NK-T (iNKT) cells, innate-like lymphocyte cells (ILCs), and mucosal-associated invariant T (MAIT) cells (2). These interactions are mediated and sustained by different cytokine pathways, and IL-23 is considered one of the most relevant early disease mediators as supported by the IL-23 signature in synovial tissue correlating with the degree of synovitis (3). IL-23 is secreted by activated macrophages, monocytes, and dendritic cells at distant sites (e.g., skin and gut) (3) and elicits the production of IL-17 in target cells, including T cells, γδ T cells, ILC3, and MAIT CD8+ cells (4).

Apremilast is a targeted synthetic disease-modifying antirheumatic drug (tsDMARD) that has proven to be effective in the treatment of PsA (5–7) by inhibiting phosphodiesterase 4 (PDE4) and preventing the hydrolysis of cyclic adenosine monophosphate (cAMP) to AMP to ultimately modulate multiple signaling pathways (8) in T cells, monocytes, and macrophages (9–11). The inhibition of PDE4 downregulates the production of pro-inflammatory cytokines, such as TNF-α, IFN-γ, and IL-23, from peripheral blood monocytes and T cells, while increasing IL-10 (12–15).

We herein report *ex vivo* data on the effects of apremilast on cytokine expression by peripheral blood mononuclear cells in patients with PsA and the correlation of baseline immunological markers with the different clinical responses to PDE4 inhibition. The cytokine analyses were performed in (a) monocyte-derived macrophages, representatives of the myeloid compartment; (b) conventional T cells, as emblems of adaptive immunity; (c) innate-like lymphocyte subsets (ILCs, MAIT cells, γδ T cells, NK, and NKT-like cells).

## Materials and methods

### Subjects

Patients with PsA fulfilling the CASPAR criteria (16) were enrolled if they had active disease according to the Disease Activity of Psoriatic Arthritis (DAPSA) index (17) and had not received previous bDMARD or tsDMARD therapy. Patients started apremilast 30 mg BID according to standard practice and current recommendations and were then followed up for 4 months. Concomitant therapies with glucocorticoids (maximum daily dosage 10 mg of prednisone equivalents) or methotrexate (maximum dosage 15 mg weekly) were allowed for patients at a stable dose for the previous 3 months. After 4 months, patients with PsA were classified as responders to apremilast if they had reached minimal disease activity (MDA) (18, 19). Patients with knee osteoarthritis (OA) were enrolled as controls.

Peripheral blood samples (30 mL) were collected for all subjects at baseline; for patients with PsA, peripheral blood sample collection was repeated also 7 days, 1 month, and 4 months of apremilast treatment.

### Monocyte isolation and differentiation

Patient peripheral blood mononuclear cells (PBMCs) were isolated by Ficoll-Paque density gradient centrifugation. Cells were subsequently washed twice with phosphate-buffered saline (PBS), counted in Trypan blue excluding dead cells (>90% viable), resuspended in freezing medium containing 90% fetal bovine serum (FBS) and 10% dimethyl sulfoxide (DMSO), aliquoted into cryovials and stored in Mr. Frosty, kept at −80°C for 24 h, and, finally, stored in liquid nitrogen cryo-containers until used.

PBMCs were thawed and left resting in complete RPMI medium for 24 h in low binding plates, inside the incubator. The following day, PBMCs were magnetically labeled with CD14+ beads (Miltenyi Biotec) and enriched with MS columns placed in OctoMACS (Miltenyi Biotec) according to manufacturer protocol to isolate monocytes by positive selection. Monocytes were recounted, and 0.2–0.3 × 10^6^ eluted CD14+ cells were seeded in a 24-well flat-bottom plate in complete media with 20 ng/ml of GM-CSF (Miltenyi Biotec) for M1 differentiation or 20 ng/ml of M-CSF (Miltenyi Biotec) for M2 differentiation, for 3 days. Then, 20 ng/ml of IFN-γ (BioLegend) and LPS (Miltenyi Biotec) for M1 and 20 ng/ml of IL-4 and IL-13 for M2 were added, respectively, and cells were cultured for other 4 days (**Supplementary Figures S1A, B**) (20). Afterward, supernatants were collected, and cells were lysate by adding 1 ml RNAzol RT RNA Isolation Reagent (#R4533 Sigma-Aldrich) and then mixed with gentle pipetting. The lysate was transferred to 2-ml Eppendorf tubes, incubated at room temperature for 5 min, to allow the complete dissociation of nucleoprotein complexes, and then stored at −70°C until RNA isolation was performed. Supernatants were also stored at −70°C until further use.

### Cytokine gene and protein expression in M1 and M2 monocyte-derived macrophages

To isolate the total RNA of M1 and M2, frozen lysate samples were thawed at room temperature and isolated according to the manufacturer’s protocol. Shortly, we added RNase-free water to precipitate DNA, proteins, and polysaccharides, which were then centrifuged, and supernatants were collected and transferred to a new tube. Isopropanol was used to precipitate total RNA, and 75% ethanol was used for washing pellet. The pellet of RNA was resuspended in RNase-free water, and the quality of extracted RNA was measured by a NanoDrop spectrophotometer. The RNA samples were then stored at −70°C until used.

We retro-transcribed RNA samples using high-capacity cDNA Reverse Transcription Kit with RNase Inhibitor (Applied Biosystems™ by Thermo Fisher). From each sample, different volumes of RNA were transferred in DNA/RNA/protein-free tubes (0.2 ml), and a retro-transcription mix was added following the kit instructions, to obtain 1 μg of DNA each. Custom TaqMan TM Gene Expression Assay (Thermo Fisher) was used for the qPCR to assess gene expression following the manufacturer’s protocol. GAPDH was used as housekeeping gene as control in all the reverse transcription (RT)-qPCR experiments. For M1, we analyzed IL-1β, IL-23, and TNF-α expression, while for M2, we analyzed IL-10, IL-1Ra, and TGF-β.

Supernatants from each experimental condition of M1 and M2 were analyzed for the same cytokines, i.e., IL-1β, IL-23, and TNF-α, and IL-10, IL-1Ra, and TGF-β, respectively, using commercially available ELISA kits (DuoSet, R&D Systems) based on the manufacturer’s protocols; all analyses were performed in duplicate. Shortly, the specific antibody for the particular cytokine was coated on a 96-well plate. Once standards and samples were pipetted into the wells, the protein present in the supernatant bound to the well tanks to the immobilized antibody, and then the HRP-conjugated streptavidin was pipetted to the wells. The wells were washed multiple times at specific steps of each ELISA. A TMB substrate solution was added to the wells, and color developed in proportion to the amount of the cytokine bound. Once the stop solution was added, this changed the color from blue to yellow, and the intensity of the color was measured at 450 nm. Serum levels of IL-23 and IL-17 were measured by ELISA (Invitrogen) in 20 patients with PsA before starting apremilast treatment.

### Lymphocyte phenotype and cytokine expression

T and innate-like cell phenotype and cytokine production of peripheral blood mononuclear cells (IL-17, IFN-γ, IL-10, and IL-9) was analyzed by flow cytometry, as detailed hereafter. Shortly, 1 × 10^6^ cells/well were seeded in a round-bottom 96-well under two conditions: unstimulated and stimulated with PMA 50 ng/ml and ionomycin 1 µg/ml and BFA for 2 h before staining in different time points of the therapy with apremilast.

Mononuclear cells were, thus, collected and stained at room temperature for membrane markers, 37°C for receptors and after permeabilization/fixing for intracellular markers using fluorochrome-conjugated monoclonal antibodies (mAbs) for identifying T cells (CD3+ CD56− γδTCR−), NKT-like cells (CD3+ CD56+ γδTCR−), NK cells (CD3− CD56+), γδ T cells (CD3+ CD56− γδTCR+), MAIT cells (CD3+ Vα7.2TCR+ CD161+), ILC1 (lineage marker cocktail (CD3, CD14, CD16, CD19, CD20, CD56)-CD127+CRTH2−cKit−), ILC3 (lineage marker cocktail (CD3, CD14, CD16, CD19, CD20, CD56)-CD127+CRTH2−cKit+), CD69, IL-17, IL-9, IFN-γ, and IL-10.

The T cell/NKT-like/γδT, NK cell panel included BV650 anti-human CD3, BV786 anti-human CD8, anti-human PE-cy7 CD56, anti-human γδ TCR APC, FITC anti-human IFN-γ, anti-human IL-9 PE-CFS594, and anti-human IL-10 BV421.

The MAIT cell panel included BV650 anti-human CD3, BV570 anti-human CD4, BV786 anti-human CD8, PE-Cy5 anti-human CD161, FITC anti-human Vα7.2TCR, PerCP-Cy5.5 anti-human CD45RO, PE anti-human IL-17, PE-CF594 anti-human CCR7, and PE-Cy7 anti-human CD69.

The ILC panel included lineage marker in APC, BV421 anti-human BV421, PE-cy5 anti-human c-kit, BV605 anti-human CRTH2, PE anti-human IL-17, FITC, and anti-human IFNγ.

AquaZombie was used as live–dead marker in all panels.

### Statistical analysis

Continuous variables are presented as means and standard deviation (SD) or medians and interquartile range (IQR), according to the data distribution. These variables were compared by t-test or Mann–Whitney non-parametric test for unpaired samples, while t-test or Wilcoxon non-parametric test was used for paired samples. The statistical analysis was performed with GraphPad Prism (version 10); *p*-values < 0.05 were considered statistically significant.

## Results

### Patient characteristics and clinical response to apremilast

A total of 23 patients with PsA (43% women) were enrolled with a median age of 56 years (IQR 47–61), with a median disease duration of 48 months (IQR 24–120). There were 14 out of 23 patients who had skin psoriasis (61%) and 6 (26%) with nail disease. Relevant clinical and laboratory parameters included a median tender joint count (TJC) of 2 (IQR 0–5), a swollen joint count (SJC) of 1 (IQR 0–2), C-reactive protein content of 0.41 mg/dL (IQR 0.17–0.50), and DAPSA content of 15 (IQR 10.7–17.5). Of note, methotrexate was being taken at a stable dose by 6/23 (26%) patients (median 10 mg every week, IQR 7.5–12.5), and 3/23 (13%) were on systemic glucocorticoids (median daily dose 7.5 mg, range 5–10). There were 20 patients with knee OA (median age 61 years, IQR 57–68; 10/20 women) who were enrolled as controls. Baseline characteristics of patients and controls are reported in **Supplementary Table S1**.

At the 4-month follow-up, a significant reduction in TJC [0 (IQR 0–2)], SJC [0 (IQR 0–1)], and DAPSA [7 (3.1–17.2)] was observed in the whole cohort of patients with PsA, while serum CRP remained stable from the baseline values [0.5 mg (IQR 0.0–0.7)] (**Supplementary Table S1**). The use of methotrexate did not influence these observations when compared with apremilast monotherapy (*data not shown*).

There were 17 out of 23 patients with PsA (74%) who were classified as responders, likely due to the limited prevalence of enthesitis and the high frequency of oligoarticular involvement; very low disease activity (VLDA) was reported in 6/23 (26%) patients. Of note, response to apremilast was independent of baseline clinical and biochemical parameters, nor did concurrent therapies influence the response to the drug (**Supplementary Table S1**).

### Inflammatory cytokine expression in monocyte-derived M1 macrophages

**Figure 1:**
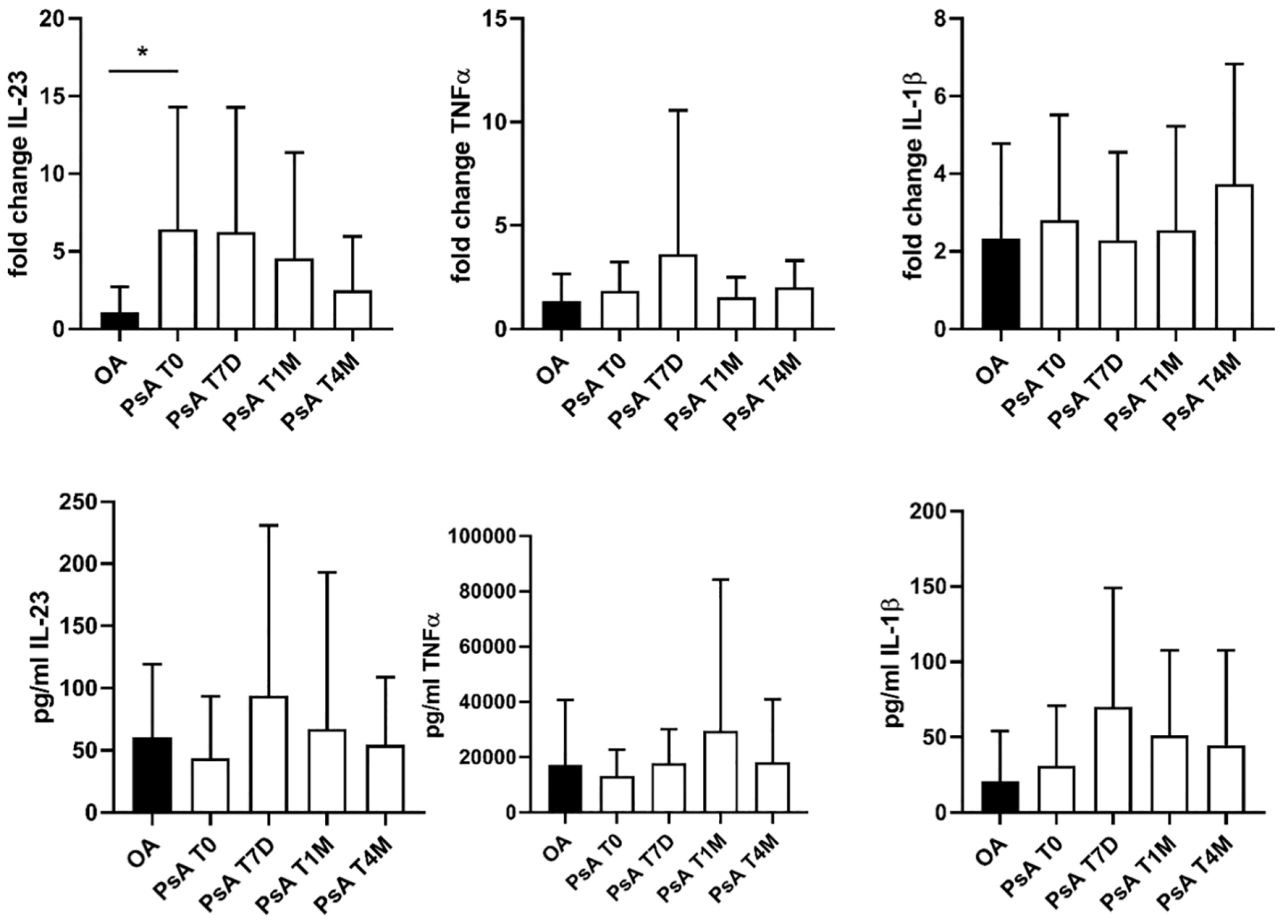
Gene expression (reported as fold change) and supernatant levels (pg/mL) of inflammatory cytokines (IL-23, TNF-α, and IL-1β) from monocyte-derived M1 macrophages. Data are reported for OA controls and patients with PsA at the baseline condition (T0), after 7 days (T7D), 1 month (T1M), and 4 months (T4M) of treatment with apremilast. *p < 0.05 comparing OA controls and PsA patients at baseline.

Compared with OA, the gene expression of IL-23 in M1 macrophages was higher in PsA at baseline (*p* = 0.003) and showed a progressive decrease after 4 months of apremilast therapy (**Figure 1**; **Supplementary Table S2**). The gene expression and supernatant levels of TNF-α and IL-1β did not differ significantly between PsA and OA, nor did they significantly change during the follow-up with apremilast treatment (**Figure 1**; **Supplementary Table S2**).

**Figure 2:**
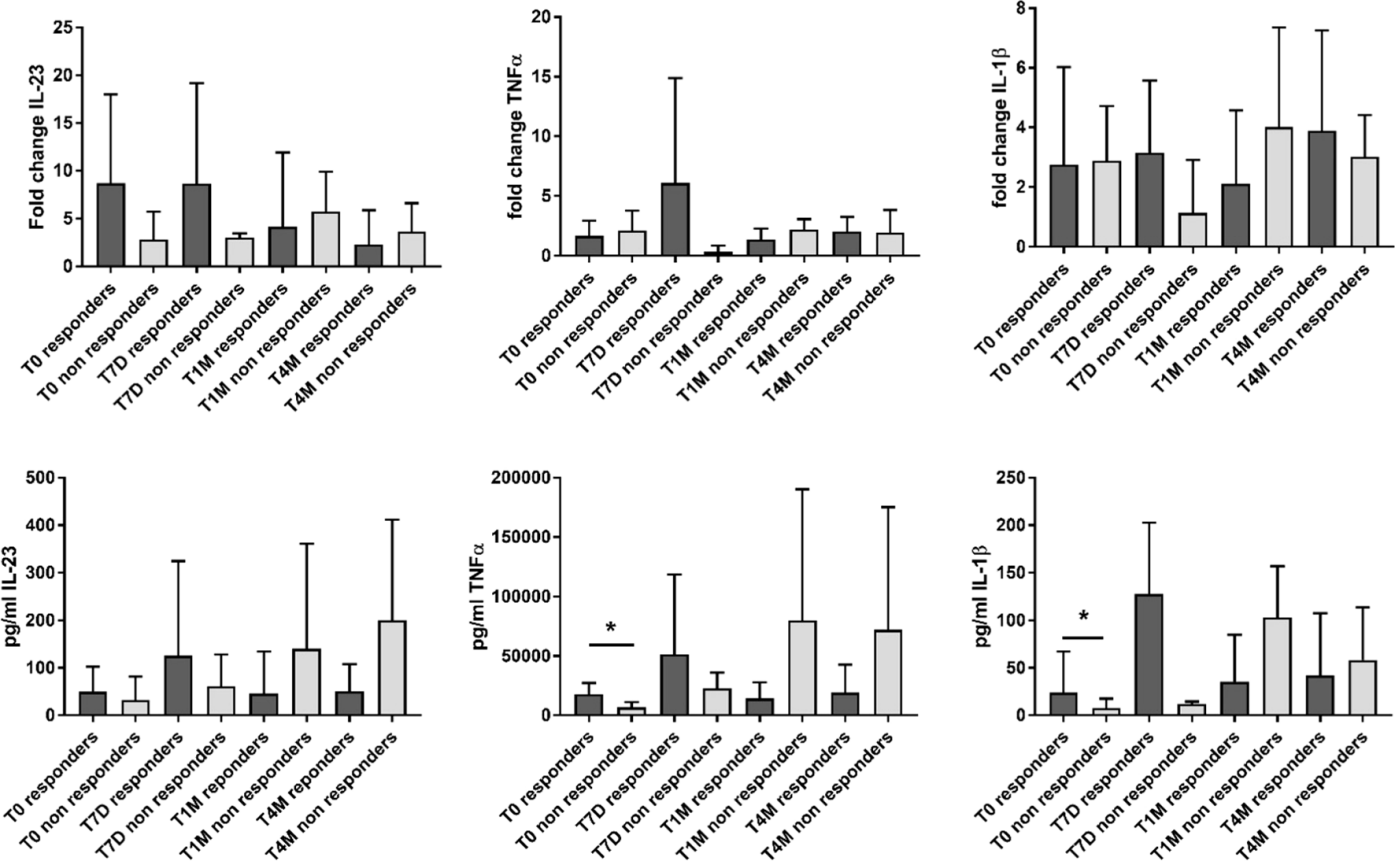
Gene expression (reported as fold change) and supernatant levels (pg/mL) of inflammatory cytokines (IL-23, TNF-α, and IL-1β) from monocyte-derived M1 macrophages in patients responding and not responding to apremilast. Data are reported for the baseline condition (T0), after 7 days (T7D), 1 month (T1M), and 4 months (T4M) of treatment with apremilast. *p < 0.05 comparing responders vs. non-responders.

When stratifying the analysis based on the clinical response to apremilast, responders showed higher levels of inflammatory cytokines at baseline compared with non-responders. This myeloid inflammatory signature was driven by higher levels of IL-23 (p not significant), TNF-α (*p* = 0.03), and IL-1β (*p* = 0.03) (**Figure 2**; **Supplementary Table S3**). However, during the 4-month follow-up, only the expression of IL-23 showed a trend toward a decrease in responders (**Figure 2**; **Supplementary Table S3**). On the other hand, increased supernatant levels of the inflammatory cytokines were observed in non-responders at the 4-month evaluation (*p* not significant) (**Figure 2**; **Supplementary Table S3**).

### Regulatory cytokine expression in monocyte-derived M2-macrophages

At baseline, the expression of regulatory molecules from M2 macrophages was enhanced in OA controls compared with PsA; this was particularly evident for IL-10, despite not reaching significance (**Supplementary Figure S2**; **Supplementary Table S4**). A significant increase in the levels of TGF-β was observed in the supernatant during treatment with apremilast (*p* = 0.023) (**Supplementary Figure S2**; **Supplementary Table S4**).

No significant change in regulatory cytokines was observed when stratifying patients with PsA according to their clinical response to apremilast. However, IL-10 tended to decrease in responders, while both IL-10 and TGF-β tended to increase in non-responders (**Supplementary Figure S3**; **Supplementary Table S5**).

### Baseline serum IL-23 and the response to apremilast

**Figure 3:**
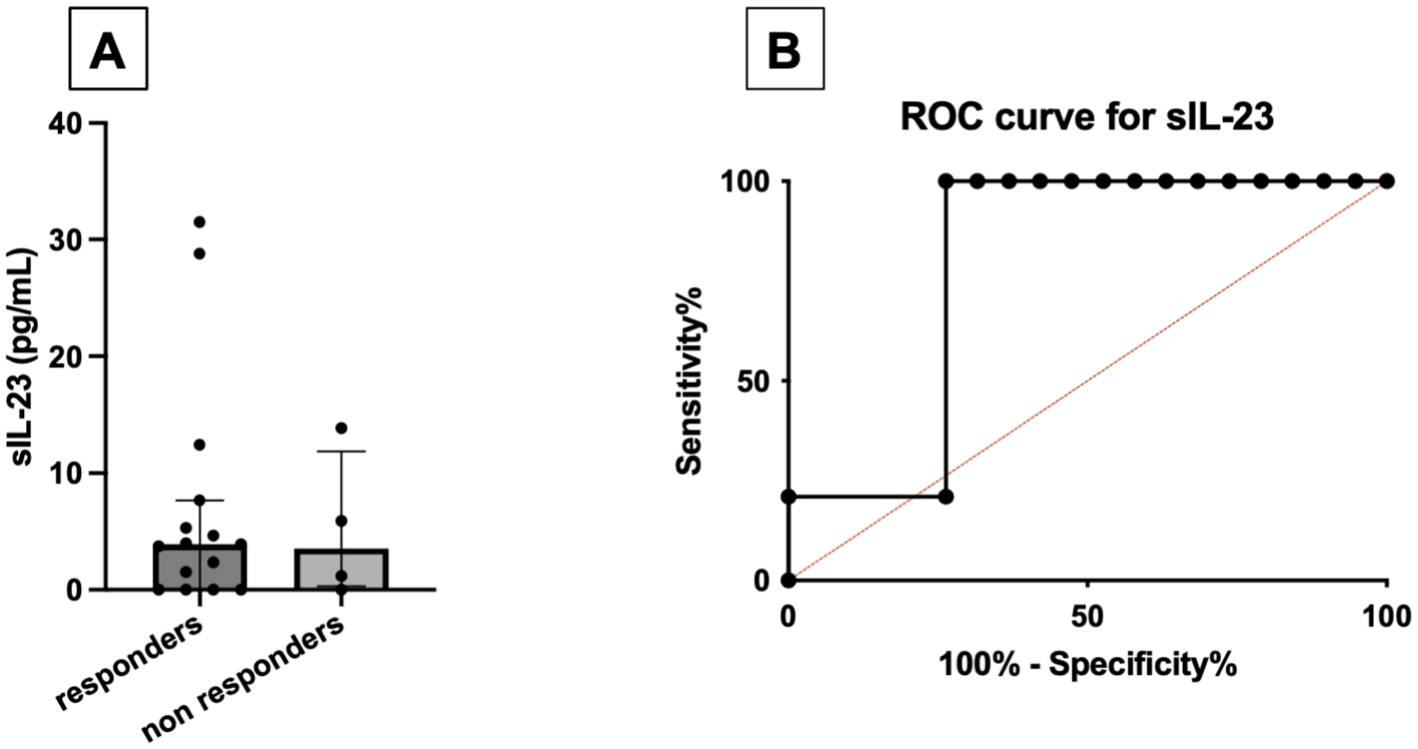
Comparison between baseline serum levels of IL-23 (pg/mL) between responders and non-responders to apremilast treatment **(A)**. ROC curve for baseline serum IL-23, using the cutoff of 1.4 pg/mL **(B)**.

Baseline levels of serum IL-23 were higher in responder PsA [3.93 pg/mL (0.0–7.7)] compared with non-responders [1.2 pg/mL (0.0–9.9)]. A baseline serum concentration of IL-23 exceeding 1.4 pg/mL predicted the response to apremilast at 4 months with moderate accuracy (AUC_ROC_ 0.79; sensitivity 100%, specificity 68%) (**Figure 3**).

Of note, serum levels of IL-23 were independent of other demographic or clinical variables, including sex, age, disease duration, TJC and SJC, DAPSA, and CRP values. Moreover, the use of methotrexate was not associated with serum levels of IL-23 (**Supplementary Table S6**). Excluding patients on concomitant methotrexate therapy, responders still had higher values of serum IL-23 [3.93 pg/mL (IQR 0.00–5.14)] compared with non-responders [1.17 pg/mL (IQR 0.58–3.54); *p* = 0.6]. No difference was still found when excluding patients on glucocorticoid therapy [3.95 pg/mL (1.53–7.69) vs. 5.91 pg/mL (0.00–13.9); p = 0.75] (*data not shown*). Serum levels of IL-17 did not differ among patients responding and not responding to apremilast (*data not shown*).

### Modulation of T and innate-like lymphocytes by apremilast

**Figure 4:**
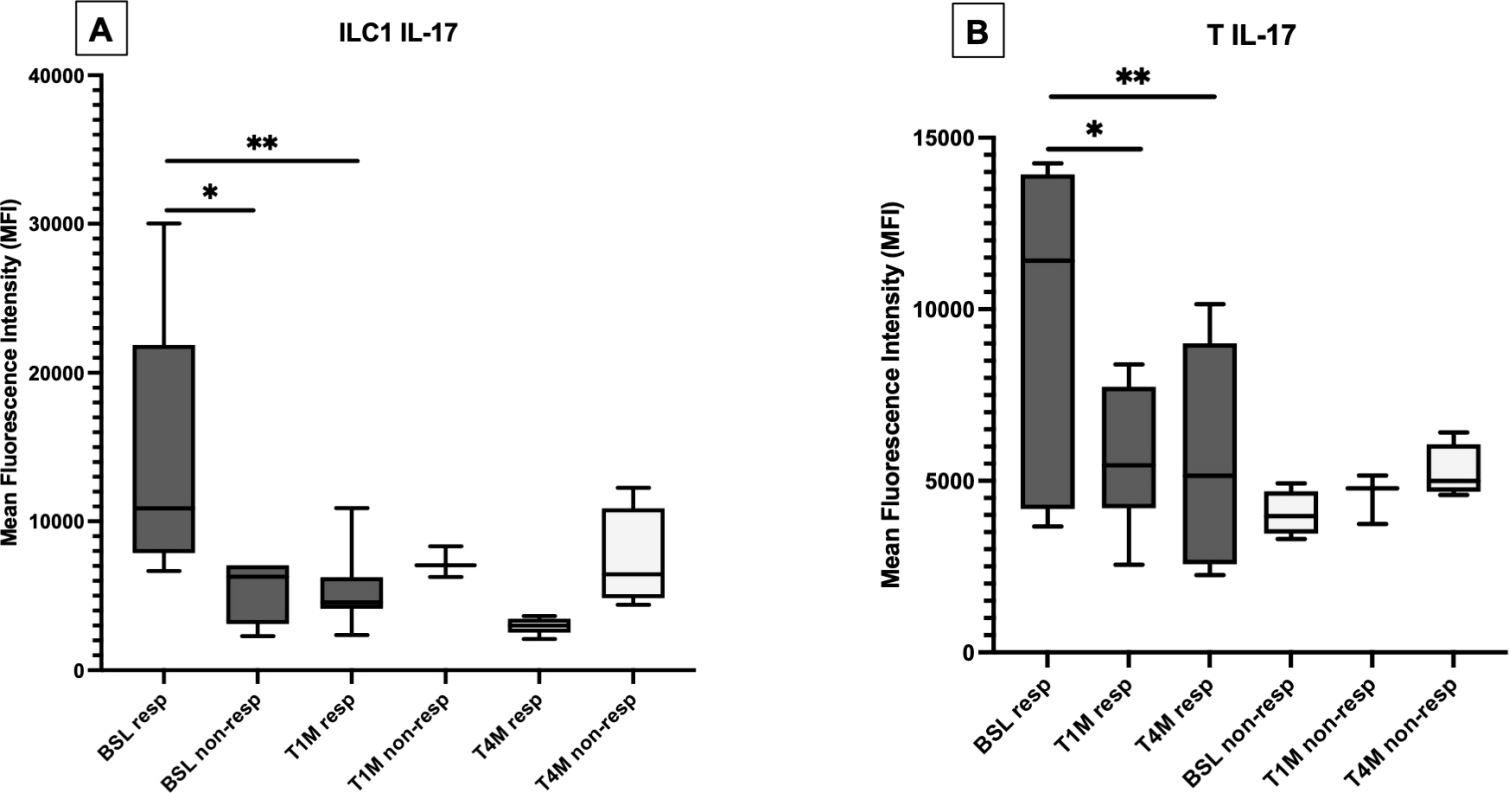
Production of IL-17 from ILC1 **(A)** and T cells **(B)** at baseline (BSL), after one (T1M) and 4 months (T4M) of apremilast treatment in responders and non-responders. For Panel A: *p < 0.05 comparing baselines for responders vs. non-responders at T4M. **p < 0.05 comparing baseline and T1M. For Panel B: *p < 0.05 comparing baseline and T1M; **p < 0.05 comparing baseline and T4M.

At baseline, the production of IL-17 was enhanced in ILC1, ILC3, and MAIT cells from patients with OA compared with PsA (**Supplementary Tables S7**, **S8**); however, the expression of this cytokine was higher in responders, compared with non-responders. Treatment with apremilast reduced the production of IL-17 from ILC1 (**Figure 4A**; **Supplementary Table S9**) and from T cells (**Figure 4B**) in responders, with no significant effect on MAIT cells (**Supplementary Table S10**).

**Figure 5:**
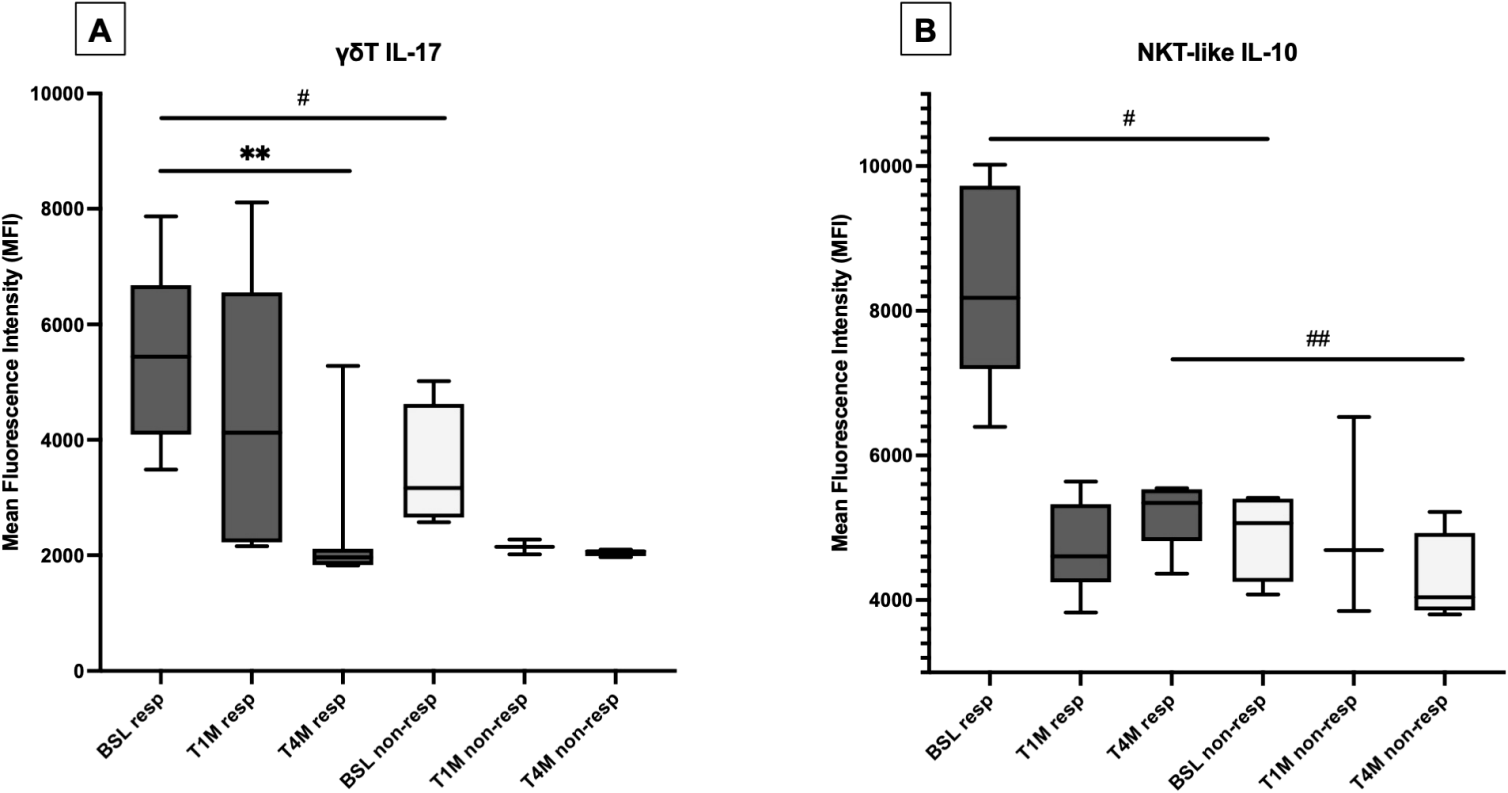
Production of IFN-γ from γδ T cells at baseline (BSL) was higher in responders compared with non-responders (^#^p < 0.05) and significantly decreased after 4 months (T4M) of treatment with apremilast only in responders (**p < 0.05) **(A)**. Production of IL-10 from NKT-like cells was higher in responders compared with non-responders at baseline (^#^p < 0.05) and at T4M (^##^p < 0.05) **(B)**.

At baseline, γδ T cells produced more IFN-γ in the peripheral blood of patients with PsA compared with OA (**Supplementary Table S11**). The production of this cytokine was more abundant in patients responding to apremilast, being sustained by different lymphocyte subsets, and significantly impaired by apremilast in γδ T cells (**Figure 5A**; **Supplementary Table S12**).

At baseline, IL-10 was more expressed from γδ T cells, NK, and NKT-like cells of patients with PsA compared with OA (**Supplementary Table S11**), particularly in responders (**Figure 5B**). A trend toward reduction was observed with apremilast in both responders and non-responders, while no relevant change was described regarding IL-9 (**Supplementary Table S12**).

## Discussion

Current recommendations for the management of PsA do not support the use of any predictor of the response to treatments, including the small-molecule apremilast (1, 21). Despite demonstrating efficacy in randomized clinical trials and real-life studies (22), the 3-year retention rate of apremilast is approximately 60%, with half of discontinuations due to primary or secondary inefficacy (22). As confirmed in our cohort, no clinical or conventional laboratory tool can predict the response to apremilast in patients affected by PsA (23).

We herein propose that serum IL-23 may predict the response to apremilast in patients with PsA, with excellent sensitivity and acceptable specificity. Although further validation on independent prospective cohorts is necessary to establish more specific cutoff values, the significance of our results is underscored by the fact that they remained unaffected by concurrent therapy with methotrexate. In agreement with our results, it was previously demonstrated that serum IL-23 does not correlate with the clinical and laboratory parameters of disease activity in patients with spondyloarthritis (24–26). In patients with PsA, higher serum levels of IL-23-related cytokines (i.e., IL-22 and IFN-γ) correlate with the response to guselkumab, an anti-IL-23 agent (27, 28), while conflicting results have been reported on ustekinumab, an inhibitor of both IL-23 and IL-12 (29).

We further demonstrated that serum IL-23 reflects a myeloid inflammatory signature sustained by macrophage activation. Compared with OA controls, monocyte-derived macrophages from patients with PsA exhibited higher expressions of IL-23, IL-1β, and TNF-α. Such inflammatory signature was more pronounced in patients responding to apremilast, in whom it was modulated by the drug, compared with non-responders, in which it increased throughout the follow-up. Tissue-resident innate immune cells, including macrophages and dendritic cells, are indeed primary sources of IL-23 (30). The production of IL-23 from myeloid tissue-resident cells can elicit a prompt response in innate-like cells, such as γδ T cells and ILCs, that secrete large amounts of pathogenic inflammatory cytokines in a “trained immunity” model (31–33).

In our study, we found that γδ T cells are the major producers of IFN-γ in patients with PsA, similar to what was previously reported in spondyloarthritis (34, 35). While γδ T cells have been largely explored in PsA for their production of IL-17 (36, 37), these cells are also sources of IFN-γ under both physiological and pathological conditions (38). Of relevance, we found that the production of IFN-γ by γδ T lymphocytes of patients with PsA is strikingly modulated by apremilast in responders but not in non-responders, and the effect is sustained during the follow-up. This observation implements previous evidence on the role of apremilast in modulating inflammatory cytokine production from innate-like cells, such as the NKT population, as well as classical T lymphocytes (39).

Apremilast led to a significant reduction in the production of IL-17 by ILC1 and conventional T cells in responder patients, but not in ILC3 and MAIT cells, possibly due to the lack of IL-9 inhibition, being IL-9 involved in IL-23 independent IL-17 production in innate immune cells (40).

While our data provide insights into both the pathogenesis of PsA with relevant clinical–pathophysiological correlates, we are also aware of the limitations of our study. First, the small sample size limits the generalizability of our findings; our results should be confirmed on larger patient cohorts, particularly for the validation of serum IL-23 as a biomarker to predict the response to apremilast. Second, our cohort was predominantly composed of patients with oligoarthritis, while enthesitis and dactylitis were less represented, and the disease duration was heterogeneous at the time of enrollment. Similarly, the prevalence of skin psoriasis reported in our cohort was lower than that reported in literature, possibly due to the sample size and to the strict collaboration with the dermatology department which function as the leading providers of treatment in patients with predominant skin involvement, albeit in full collaboration with our Department. Third, concurrent treatment with systemic glucocorticoids might have partially influenced our results. Despite that data on serum levels of IL-23 remained unchanged after removing patients taking concurrent therapies, validation on a larger cohort of naïve patients (vs. experienced to other drugs) would serve as a proof of concept. Fourth, responders and non-responders to apremilast were defined *bona fide* based on previous literature, but more homogeneous response criteria would be of use. Indeed, according to the adopted definition, we observed a high prevalence of responders to apremilast, while only 26% reaching VLDA, which is comparable with previous studies. Such heterogeneity may reflect limitations including the small sample size, the predominance of oligoarticular phenotype in patients treated with apremilast also following the EULAR recommendations (21), the low prevalence of enthesitis and dactylitis, and the less strict response criteria. Finally, while we assessed the changes induced by apremilast on peripheral blood cells, confirmation on synovial fluid and/or tissue samples of synovium or enthesis would tailor the precision medicine approach to the treatment of patients with PsA. In particular, it would be relevant to study how apremilast modulates the expression of IL-23 on synovial/entheseal macrophages. Last, we may only hypothesize that the partial decoupling between IL-23 and IL-17 in some types of innate immune cells should be further investigated, even if it could be possibly due to the lack of IL-9 inhibition, with IL-9 being involved in IL-23 independent of IL-17 production.

In conclusion, our data support the immunological bases of apremilast response in PsA. In fact, we report that an IL-23 signature predicts the response to apremilast and characterizes monocyte-derived M1 macrophages in patients with PsA, and high baseline levels of all myeloid cytokines (IL-1β, TNF-α, and IL-23) are associated with the response to apremilast. A decreased expression of pro-inflammatory cytokines was observed in monocyte-derived macrophages (IL-23), ILC1 and T cells (IL-17), and γδ T cells (IFN-γ) of patients with PsA responding to apremilast, while the lack of response was linked to a progressive increase in the inflammatory signature.

## Data Availability

The raw data supporting the conclusions of this article will be made available by the authors, without undue reservation.
