# Supplementary material for: Serum IL-23 levels reflect a myeloid inflammatory signature and predict the response to apremilast in patients with psoriatic arthritis
Source: Front Immunol. 2024 Dec 4;15:1455134. doi: 10.3389/fimmu.2024.1455134 (PMC11652657; doi:10.3389/fimmu.2024.1455134)

**Supplementary Table 1.** Characteristics of controls with knee OA and patients with PsA at baseline and after 4 months of treatment with apremilast (T4M); * p < 0.05 comparing PsA between baseline and T4M. The comparison between baseline clinical and clinimetric indexes between responders and non-responders to apremilast is also reported, but no significant p-values have been detected.

|  | **OA**  **(n = 20)** | **PsA baseline**  **(n = 23)** | **PsA T4M**  **(n = 23)** | **Baseline values in responders**  **(n = 17)** | **Baseline values in non-responders**  **(n = 6)** |
| --- | --- | --- | --- | --- | --- |
| Age (years) [median (IQR)] | 61 (57-68) | 56 (47-61) |  | 56 (48-61) | 54 (46-62) |
| Female subjects [n (%)] | 10/20 (50%) | 10/23 (43.5) |  | 6/17 (35) | 4/6 (67) |
| TJC [median (IQR)] | n.a. | 2 (0-5) | 0 (0-2)* | 1 (0-5) | 2 (0-4) |
| SJC [median (IQR)] | n.a. | 1 (0-2) | 0 (0-1)* | 1 (0-2) | 1 (0-1) |
| CRP (mg/dL) [median (IQR)] | n.a. | 0.41 (0.17-0.5) | 0.5 (0.0-0.7)* | 0.5 (0.13-0.5) | 0.5 (0.25-0.95) |
| DAPSA [median (IQR)] | n.a. | 15 (10.7-17.5) | 7 (3.1-17-2)* | 13 (9.4-21.1) | 15 (14.5-17.1) |
| PGA [median (IQR)] | n.a. | 50 (30-70) | 20 (10-45)* | 50 (20-70) | 50 (40-60) |
| VAS pain, mm [median (IQR)] | n.a. | 55 (25-75) | 15 (5-30)* | 55 (25-75) | 50 (45-65) |
| PhGA [median (IQR)] | n.a. | 40 (30-45) | 15 (5-45)* | 45 (25-45) | 45 (40-60) |
| PsO [n (%)] | n.a. | 14/23 (61) |  | 11/17 (65) | 3/6 (50) |
| Nail psoriasis [n (%)] | n.a. | 6/23 (26.1) |  | 4/17 (24) | 2/6 (33) |
| Concomitant MTX treatment [n (%)] | n.a. | 6/23 (26.1) |  | 3/17 (17.6) | 3/6 (50.0) |
| Concomitant glucocorticoid treatment [n (%)] | n.a. | 3/23 (13.0) |  | 1/17 (5.88) | 2/6 (33.3) |

*Legend* – TJC: tender joint count; SJC: swollen joint count; CRP: C-reactive protein; DAPSA: Disease Activity in PSoriatic Arthritis; PsO: psoriasis; MTX: methotrexate; n.a.: not applicable; PGA: patient general assessment of disease activity; PhGA: physician general assessment of disease activity; T4M: after four months of treatment with apremilast; VAS pain: visual analogical scale of pain.

**Supplementary Table 2.** Gene expression (reported as fold change) and supernatant levels (pg/mL) of inflammatory cytokines (IL-23, TNF-α, and IL-1β) from monocyte-derived M1 macrophages. Data are reported for OA controls and patients with PsA at the baseline condition (T0), after 7 days (T7D), one month (T1M) and 4 months (T4M) of treatment with apremilast. Sub-analysis on patients on apremilast monotherapy is reported.

* p < 0.05 comparing OA controls and PsA patients at baseline.

|  | **OA controls** | **PsA T0** | **PsA T7D** | **PsA T1M** | **PsA T4M** |
| --- | --- | --- | --- | --- | --- |
| **All patients (n = 23)** | | | | | |
| IL-23 expression | 1.09 (1.63) | 6.43 (7.88)* | 6.24 (8.05) | 4.56 (6.82) | 2.50 (3.46) |
| IL-23 ELISA | 89.0 (101.1) | 43.9 (49.6) | 94.1 (137.1) | 67.5 (125.8) | 54.4 (54.5) |
| TNF-α expression | 1.36 (1.30) | 1.86 (1.38) | 3.62 (6.96) | 1.56 (0.95) | 2.03 (1.28) |
| TNF-α ELISA | 17262 (23479) | 13252 (9483) | 17964 (12210) | 29420 (54777) | 18285 (22854) |
| IL-1β expression | 2.33 (2.46) | 2.80 (2.72) | 2.29 (2.26) | 2.55 (2.67) | 3.74 (3.10) |
| IL-1β ELISA | 20.6 (33.8) | 31.1 (39.8) | 70.1 (79.1) | 51.1 (56.4) | 44.7 (62.9) |
| **Apremilast monotherapy (n = 14)** | | | | | |
| IL-23 expression | 1.09 (1.63) | 7.32 (10.5)* | 4.83 (4.06) | 5.04 (8.12) | 1.75 (2.09) |
| IL-23 ELISA | 89.0 (101.1) | 27.0 (32.6) | 130.5 (195.1) | 44.4 (101.7) | 31.4 (29.3) |
| TNF-α expression | 1.36 (1.30) | 1.27 (0.94) | 1.77 (1.81) | 1.69 (1.11) | 1.99 (1.29) |
| TNF-α ELISA | 17262(23479) | 17800(10467) | 15777(7365) | 14120(15364) | 16851(24798) |
| IL-1β expression | 2.33 (2.46) | 4.08 (3.42) | 3.23 (2.95) | 1.90 (2.02) | 4.36 (3.18) |
| IL-1β ELISA | 20.6 (33.8) | 46.5 (47.6)* | 116.5 (94.1) | 37.1 (55.9) | 39.0 (58.2) |

**Supplementary Table 3.** Gene expression (reported as fold change) and supernatant levels (pg/mL) of inflammatory cytokines (IL-23, TNF-α, and IL-1β) from monocyte-derived M1 macrophages in patients responding and not responding to apremilast. Data are reported for the baseline condition (T0), after 7 days (T7D), one month (T1M) and 4 months (T4M) of treatment with apremilast.

* p < 0.05 comparing responders vs. non-responders.

|  |  | **PsA T0** | **PsA T7D** | **PsA T1M** | **PsA T4M** |
| --- | --- | --- | --- | --- | --- |
| IL-23 expression | Responder (n = 17) | 8.68 (9.31) | 8.65 (10.6) | 4.13 (7.79) | 2.26 (3.60) |
|  | Non-responder (n = 6) | 2.83 (2.89) | 3.04 (0.42) | 5.72 (4.18) | 3.64 (2.96) |
| IL-23 ELISA | Responder (n = 17) | 50.4 (52.3) | 126.1 (198.7) | 45.7 (89.2) | 50.8 (57.3) |
|  | Non-responder (n = 6) | 32.5 (49.5) | 62.0 (66.5) | 140.3 (220.8) | 79.35 (24.34) |
| TNF-α expression | Responder (n = 17) | 1.68 (1.27) | 6.10 (8.81) | 1.37 (0.92) | 2.04 (1.21) |
|  | Non-responder (n = 6) | 2.14 (1.65) | 0.33 (0.53) | 2.21 (0.86) | 1.97 (1.87) |
| TNF-α ELISA | Responder (n = 17) | 17844 (9629)* | 51346(67368) | 14277 (13696) | 19007 (23667) |
|  | Non-responder (n = 6) | 6824 (4435)* | 23000(13106) | 79899(110409) | 72112(103168) |
| IL-1β expression | Responder (n = 17) | 2.75 (3.28) | 3.16 (2.41) | 2.11 (2.47) | 3.89 (3.38) |
|  | Non-responder (n = 6) | 2.88 (1.84) | 1.13 (1.78) | 4.02 (3.34) | 3.02 (1.40) |
| IL-1β ELISA | Responder (n = 17) | 47.7 (45.5)* | 127.9 (74.8) | 35.5 (49.4) | 41.9 (65.6) |
|  | Non-responder (n = 6) | 7.74 (9.90) | 12.2 (2.29) | 103.3 (53.6) | 58.4 (55.4) |

**Supplementary Table 4.** Gene expression (reported as fold change) and supernatant levels (pg/mL for IL-10 and TGF-β, and ng/mL for IL-1Ra) of regulatory cytokines (IL-10, TGF-β, IL-1Ra) from monocyte-derived M2 macrophages. Data are reported for OA controls and patients with PsA at the baseline condition (T0), after 7 days (T7D), one month (T1M) and 4 months (T4M) of treatment with apremilast.

* p < 0.05 comparing T0 and T4M in patients with PsA

|  | **OA controls** | **PsA T0** | **PsA T7D** | **PsA T1M** | **PsA T4M** |  |
| --- | --- | --- | --- | --- | --- | --- |
| **All patients (n = 23)** | | | | | | |
| IL-10 expression | 4.03 (9.24) | 0.62 (0.35) | 0.70 (0.36) | 1.89 (4.93) | 0.46 (0.34) |  |
| IL-10 ELISA | 91.1 (82.6) | 84.4 (79.2) | 187.2 (167.3) | 160.1 (224.5) | 77.4 (56.8) |  |
| TGF-β expression | 1.34 (1.30) | 1.01 (0.66) | 1.11 (0.54) | 0.94 (0.75) | 0.72 (0.28) |  |
| TGF-β ELISA | 578.1 (70.9) | 592.2 (199.3) | 655.2 (63.4) | 658.3 (130.4) | 656.7 (74.5)* |  |
| IL-1Ra expression | 2.62 (7.00) | 1.52 (1.11) | 2.32 (1.72) | 1.61 (1.01) | 1.46 (0.87) |  |
| IL-1Ra ELISA | 32.2 (31.6) | 25.0 (16.7) | 67.5 (86.7) | 31.3 (42.2) | 25.5 (19.6) |  |
| **Apremilast monotherapy (n = 14)** | | | | | | |
| IL-10 expression | 4.03 (9.24) | 0.56 (0.28) | 0.71 (0.36) | 0.47 (0.36) | 0.41 (0.33) |  |
| IL-10 ELISA | 91.1 (82.6) | 102.9 (79.8) | 124.4 (82.7) | 61.5 (49.7) | 66.7 (52.8) |  |
| TGF-β expression | 1.34 (1.30) | 0.72 (0.15) | 0.98 (0.51) | 0.86 (0.92) | 0.71 (0.30) |  |
| TGF-β ELISA | 578.1 (70.9) | 557.7 (274.7) | 676.8 (32.8) | 647.6 (156.2) | 666.4 (62.0) |  |
| IL-1Ra expression | 2.62 (7.00) | 1.09 (0.46) | 3.15 (2.01) | 1.32 (1.04) | 1.53 (0.96) |  |
| IL-1Ra ELISA | 32.2 (31.6) | 25.0 (16.7) | 67.5 (86.7) | 31.3 (42.2) | 25.5 (19.6) |  |

**Supplementary Table 5.** Gene expression (reported as fold change) and supernatant levels (pg/mL for IL-10 and TGF-β, ng/mL for IL-1Ra) of regulatory cytokines (IL-10, TGF-β, and IL-1Ra) from monocyte-derived M2 macrophages in patients responding and not responding to apremilast. Data are reported for the baseline condition (T0), after 7 days (T7D), one month (T1M) and 4 months (T4M) of treatment with apremilast.

|  |  | **PsA T0** | **PsA T7D** | **PsA T1M** | **PsA T4M** |
| --- | --- | --- | --- | --- | --- |
| IL-10 expression | Responder (n = 17) | 0.64 (0.29) | 0.64 (0.32) | 2.38 (5.68) | 0.40 (0.24) |
|  | Non-responder (n = 6) | 0.58 (0.46) | 0.78 (0.48) | 0.43 (0.37) | 0.73 (0.65) |
| IL-10 ELISA | Responder (n = 17) | 120.7 (83.0) | 285.0 (82.4) | 84.3 (82.4) | 66.5 (52.6) |
|  | Non-responder (n = 6) | 36.1 (27.6) | 89.5 (55.1) | 412.6 (384.2) | 148.1 (20.3) |
| TGF-β expression | Responder (n = 17) | 1.03 (0.75) | 0.92 (0.50) | 1.03 (0.77) | 0.70 (0.28) |
|  | Non-responder (n = 6) | 0.98 (0.56) | 1.35 (0.59) | 0.65 (0.72) | 0.80 (0.33) |
| TGF-β ELISA | Responder (n = 17) | 679.8 (41.1) | 681.7 (29.0) | 634.8 (141.3) | 649.3 (76.9) |
|  | Non-responder (n = 6) | 469.7 (273.0) | 628.7 (84.3) | 736.5 (14.2) | 708.7 (6.32) |
| IL-1Ra expression | Responder (n = 17) | 1.27 (0.49) | 1.40 (0.94) | 1.52 (1.10) | 1.43 (0.91) |
|  | Non-responder (n = 6) | 1.91 (1.73) | 3.54 (1.90) | 1.91 (0.69) | 1.58 (0.80) |
| IL-1Ra ELISA | Responder (n = 17) | 24.8 (18.7) | 95.9 (101.0) | 30.5 (45.5) | 25.2 (20.3) |
|  | Non-responder (n = 6) | 26.1 (0) | 10.7 (0) | 36.8 (0) | 30.2 (0) |

**Supplementary Table 6.** Comparison between clinical and laboratory parameters among PsA patients with high (≥ 1.4 pg/mL) or low (< 1.4 pg/mL) serum levels of IL-23 at baseline.

| **PsA** | **Serum IL-23 < 1.4 pg/mL (n = 7)** | **Serum IL-23 ≥1.4 pg/mL (n = 13)** | ***p*** |
| --- | --- | --- | --- |
| Female sex | 3 | 6 | >0.9 |
| Age (median) | 50 | 60 | 0.3 |
| TJC (median) | 2 | 2 | >0.9 |
| SJC (median) | 1 | 0 | 0.2 |
| CRP (median) | 0.5 | 0.2 | 0.1 |
| DAPSA (median) | 16.5 | 12.5 | 0.3 |
| MTX (n, %) | 3 | 3 | 0.66 |
| Disease duration (months) | 1 | 3 | 0.6 |

**Supplementary Table 7.** Comparison between ILC populations and cytokine production between patients with PsA and OA at baseline. * p < 0.05 comparing PsA and OA

|  | **PsA (n = 23)** | **OA (n = 20)** |
| --- | --- | --- |
| ILC1 (unstimulated) | 79.1 (53.4-86.9) | 62.3 (59.5-75.1) |
| ILC2 (unstimulated) | 2.5 (1.3-11.2) | 4.70 (1.88-7.38) |
| ILC3 (unstimulated) | 12.3 (7.0-22.6) | 20.0 (15.9-22.7) |
| ILC1 IFN-γ (%)  ILC1 IFN-γ (MFI) | 17.4 (5.8-30.4)  3250 (1969-4840) | 18.6 (17.4-23.7)  5426 (4810-5681) |
| ILC1 IL-17 (%)  ILC1 IL-17 (MFI) | 1.1 (0.9-2.3)  7858 (6663-13866) | **3.10 (2.85-3.35)***  15237 (12838-17160) |
| ILC2 IFN-γ (%)  ILC2 IFN-γ (MFI) | 0 (0-9.1)  0 (0-2029 | 0 (0-1.58)  0 (0-1364) |
| ILC2 IL-17 (%)  ILC2 IL-17 (MFI) | 2.4 (0-12.5)  1804 (0-6689) | 8.30 (7.40-14.6)  7564 (1512-11277) |
| ILC3 IFN-γ (%)  ILC3 IFN-γ (MFI) | 4.1 (3.1-5.3)  2579 (1971-3308) | 1.75 (0.25-3.48)  2281 (545-2504) |
| ILC3 IL-17 (%)  ILC3 IL-17 (MFI) | 10.6 (4.5-27.8)  3495 (1212-14799) | 9.75 (5.33-18.7)  **35424 (18893-46512)*** |

**Supplementary** **Table 8.** Comparison between MAIT and conventional T cell populations and the respective production of IL-17 between patients with PsA and OA at baseline. * p < 0.05 comparing PsA and OA

|  | **PsA (n = 23)** | **OA (n = 20)** |
| --- | --- | --- |
| MAIT | 1.8 (0.7-3.3) | 2.00 (1.13-2.5) |
| MAIT CD8+ | 77.6 (55.9-81.2) | 87.4 (84.7-88.9) |
| MAIT CD8+ eff | 78.7 (63.5-87.6) | 84.2 (82.9-86.6) |
| MAIT CD8+ CD69+ (%)  MAIT CD8+ CD69+ (MFI) | 98.8 (97.8-99.7)*  8736 (6792-13840)* | 93.6 (90.0-96.3)  5303 (4680-6145) |
| MAIT CD8+ IL-17 (%)  MAIT CD8+ IL-17 (MFI) | 1.6 (0.9-2.6)  3871 (1911-4176) | 1.30 (0.60-2.30)  4956 (3905-6789)* |
| MAIT CD8+ eff IL-17 (%)  MAIT CD8+ eff IL-17 (MFI) | - 1. (0.6-2.6)   3872 (2615-6432) | 1.05 (0.83-2.25)  4439 (4078-4996) |
| CD3+ | 78.0 (72.4-87.7) | 71.5 (63.8-75.7) |
| CD3+ IL-17 (%)  CD3+ IL-17 (MFI) | 0.9 (0.7-1.1)  4926 (3924-12779) | 0.70 (0.60-0.95)  9833 (8628-10996) |

**Supplementary Table 9.** Comparison between ILCs populations and cytokine production at baseline, after one (T1M), and four months (T4M) of treatment with apremilast in patients with PsA.

* p < 0.05 comparing T1M and baseline; ** p < 0.05 comparing T4M and baseline; # p < 0.05 comparing responder vs non-responder at baseline;

^p < 0.05 comparing responder vs non-responder at T1M; $ p < 0.05 comparing responder vs non-responder at T4M

|  |  | **Baseline** | **T1M** | **T4M** |
| --- | --- | --- | --- | --- |
| ILC1 (unstimulated) | Responder (n = 17) | 54.7 (53.4-86.9) | 76.4 (45.0-82.2) | 52.8 (33.9-76.4)^$^ |
|  | Non-responder (n = 6) | 82 (51.9-91.6) | 92 (68.1-94.7) | 87.4 (68.9-92.0) |
| ILC2 (unstimulated) | Responder (n = 17) | 5.9 (1.3-12.4)^#^ | 2.0 (1.4-3.8) | 1.9 (1.40-2.80) |
|  | Non-responder (n = 6) | 1.7 (0.9-2.3) | 0.8 (0.1-1.9) | 1.3 (0.7-3.0) |
| ILC3 (unstimulated) | Responder (n = 17) | 15.3 (7.1-22.6) | 15.5 (4.2-52.7) | 37.4 (18.2-63.0) |
|  | Non-responder (n = 6) | 8.4 (4.5-40.8) | 5.6 (4.3-5.8) | 4.4 (4.2-23.4) |
| ILC1 IFN-γ (%)  ILC1 IFN-γ (MFI) | Responder (n = 17) | 22.4 (5.8-30.8)  3250 (1969-5936) | 13.8 (5.1-18.6)*  2459 (2367-5225) | 19.2 (11.7-24.0)  5555 (3286-6301) |
|  | Non-responder (n = 6) | 14.1 (5.7-27.2)  3454 (2071-4568) | 10.9 (9.1-25.4)  3118 (3062-4522) | 9.6 (8.3-14.6)  3356 (2635-5247) |
| ILC1 IL-17 (%)  ILC1 IL-17 (MFI) | Responder (n = 17) | 1.5 (0.9-2.3)  11616 (8235-19867)^#^ | 1.60 (0.80-1.80)*  4560 (4131-6240)* | 2.90 (1.70-5.90)  3327 (2675-3636)**^$^ |
|  | Non-responder (n = 6) | - 1. (0.7-2.1)   6276 (3098-7043) | 1.8 (1.2-4)  7050 (6263-8319) | 2.2 (1.5-3.6)  6440 (4833-10871) |
| ILC2 IFN-γ (%)  ILC2 IFN-γ (MFI) | Responder (n = 17) | 0 (0-11.1)  0 (0-5951) | 0 (0-4.5)  0 (0-1381) | 11.9 (0-18.6)  3426 (0-4906) |
|  | Non-responder (n = 6) | 0 (0-0)  0 (0-0) | 0 (0-12.1)  0 (0-2082) | 0 (0-2.8)  0 (0-1429) |
| ILC2 IL-17 (%)  ILC2 IL-17 (MFI) | Responder (n = 17) | 2.4 (0-13.6)  5762 (0-10821) | 2.20 (0-5.40)  2120 (0-7826) | 41.9 (14.3-47.50)  5063 (4646-7809) |
|  | Non-responder (n = 6) | 2.7 (0-10.7)  902 (0-2262) | 4.9 (0-21.2)  4118 (0-5313) | 8.5 (1.3-15.0)  3609 (819.8-4639) |
| ILC3 IFN-γ (%)  ILC3 IFN-γ (MFI) | Responder (n = 17) | 4.1 (3.1-5.3)  2461 (1042-6321) | 3.50 (1.0-9.80)  1779 (1218-4297) | 10.4 (1.3-15.50)**  3667 (2604-4693)^$^ |
|  | Non-responder (n = 6) | 4.2 (1.3-9.7)  2581 (2316-3127) | 3.5 (3.5-10)  2421 (2299-2493) | 2.9 (0.3-4.9)  2189 (535.8-2276) |
| ILC3 IL-17 (%)  ILC3 IL-17 (MFI) | Responder (n = 17) | 17.5 (7.9-35.5)  8911 (1212-62068) | 11.1 (7.80-25.2)*  2136 (1357-22596)* | 31.9 (16.0-36.7)  23269 (18268-23836)^$^ |
|  | Non-responder (n = 6) | 4.7 (4.0-9.2)  2131 (1230-3500) | 9.2 (8.8-10.3)  3437 (2855-5664) | 7.5 (4.9-13.8)  5994 (3347-1428) |

**Supplementary** **Table 10.** Comparison between MAIT and classical T cells, and their production of IL-17 at baseline, after one (T1M), and four months (T4M) of treatment with apremilast in patients with PsA.

* p < 0.05 comparing T1M and baseline; ** p < 0.05 comparing T4M and baseline; # p < 0.05 comparing responder vs non-responder at baseline; ^p < 0.05 comparing responder vs non-responder at T1M; $ p < 0.05 comparing responder vs non-responder at T4M

|  |  | **Baseline** | **T1M** | **T4M** |
| --- | --- | --- | --- | --- |
| MAIT | Responder (n = 17) | 1.9 (0.70-3.30) | 1.40 (1.10-3.01) | 1.80 (1.6-3.7) |
|  | Non-responder (n = 6) | 1.5 (0.6-4.4) | 0.9 (0.4-1.2) | 0.8 (0.4-7.9) |
| MAIT CD8+ | Responder (n = 17) | 77.6 (55.8-81.0) | 73.7 (63.2-82.5) | 74.3 (58.5-76.5)^$^ |
|  | Non-responder (n = 6) | 78.5 (60.3-85.8) | 64.2 (47.7-78.8) | 55.3 (28.7-82.9) |
| MAIT CD8+ eff | Responder (n = 17) | 78.7 (48.0-87.6) | 66.3 (58.7-78.1) | 73.6 (65.2-88.4) |
|  | Non-responder (n = 6) | 74.1 (66.1-89.1) | 73.6 (67.3-78.1) | 65.2 (45.3-86.4) |
| MAIT CD8+ CD69+ (%)  MAIT CD8+ CD69+ (MFI) | Responder (n = 17) | 98.5 (87.2-98.8)^#^  7758 (6269-8736)^#^ | 95.3 (93.1-97.5)  5281 (4790-9049)^ | 97.9 (97.0-98.80)  7473 (4945-8763) |
|  | Non-responder (n = 6) | 99.9 (99.7-100)  17076 (12759-22561) | 98 (97.5-99.2)  13555 (10069-14581) | 95.6 (65.5-98.9)  9911 (5786-15274) |
| MAIT CD8+ IL-17 (%)  MAIT CD8+ IL-17 (MFI) | Responder (n = 17) | 2.2 (1.60-3.2)^#^  2136 (1821-4120) | 1.70 (0.80-2.10)  3993 (2738-4885)* | 1.90 (1.60-2.90)  3519 (2338-4432) |
|  | Non-responder (n = 6) | 0.9 (0.8-1.2)  4095 (3416-4383) | 2.3 (0.5-2.4)  2500 (1903-8224) | 1. (0.3-2.7)   2786 (2303-3258) |
| MAIT CD8+ eff IL-17 (%)  MAIT CD8+ eff IL-17 (MFI) | Responder (n = 17) | 1.2 (0.60-3.3)  5108 (2768-7377) | 1.30 (0.70-1.70)  4554 (2585-4994) | 1.10 (0.50-3.0)  4043 (3692-7288) |
|  | Non-responder (n = 6) | - 1. (0.7-2.3)   3019 (2331-3779) | 1.9 (0.5-2.4)  2515 (1817-6293) | 1.3 (0.5-1.8)  4667 (3011-7282) |
| CD3+ IFN-γ (%)  CD3+ IFN-γ (MFI) | Responder (n = 17) | 14.9 (9.3-19.3)  6351 (5540-7604)^#^ | 17.0 (12.2-22.3)  6758 (6442-8096)^^^ | 17.6 (15.3-22.8)^$^  7889 (7507-8948)^$^ |
|  | Non-responder (n = 6) | 14.8 (9.5-22.1)  4252 (3450-4732) | 16.9 (6.9-25.3)  4174 (3417-5001) | 9.4 (6.2-11.4)  3682 (2947-4118) |
| CD3+ IL-17 (%)  CD3+ IL-17 (MFI) | Responder (n = 17) | 0.70 (0.30-0.90)  11413 (4175-13993) | 0.80 (0.70-1.90)  5456 (4192-7741) | 1.40 (1.20-4.30)**  5150 (2564-9009)** |
|  | Non-responder (n = 6) | - 1. (1-1.4)   3964 (3458-4696) | 0.7 (0.6-0.9)  4780 (3734-5152) | 0.5 (0.3-1.5)  4994 (4679-6071) |

**Supplementary Table 11.** Comparison between T, NK, NKT-like, and γδ T cell populations and cytokine production between patients with PsA and OA at baseline. * p < 0.05 comparing PsA and OA.

|  | **PsA (n = 23)** | **OA (n = 20)** |
| --- | --- | --- |
| γδ T cells | 0.8 (0.6-1.1) | 1.00 (0.80-1.50)* |
| NK cells | 5.2 (3.5-7.9) | 9.50 (5.83-12.5) |
| NKT cells | 2.0 (1.2-2.8) | 7.45 (4.43-8.30)* |
| T cells | 78.0 (72.4-87.7) | 71.5 (63.8-75.7) |
| γδ T IFN-γ (%)  γδ T IFN-γ (MFI) | 31.9 (23.3-39.5)*  4143 (3430-6039)* | 7.65 (5.43-13.8)  1950 (1815-2199) |
| γδ T IL-9 (%)  γδ T IL-9 (MFI) | 0 (0-1.1)  0 (0-705) | 0 (0-0)  0 (0-0) |
| γδ T IL-10 (%)  γδ T IL-10 (MFI) | 1.6 (0.6-4.7)*  4727 (3345-7123)* | 0.45 (0.10-0.65)  1850 (453-1989) |
| NK IFN-γ (%)  NK IFN-γ (MFI) | 25.3 (10.1-38.8)  2730 (2173-3121) | 37.2 (32.4-42.6)  3226 (3072-3364)* |
| NK IL-9 (%)  NK IL-9 (MFI) | 0.2 (0.05-0.4)  567 (505.5-684) | 0.09 (0.07-0.10)  564 (544-691) |
| NK IL-10 (%)  NK IL-10 (MFI) | 0.4 (0.3-1.89)  4756 (3765-5520)* | 0.35 (0.30-0.40)  2647 (2418-2757) |
| NKT IFN-γ (%)  NKT IFN-γ (MFI) | 51.2 (44.5-64.3)  4878 (4157-5907) | 47.7 (42.8-55.7)  7083 (6887-7232)* |
| NKT IL-9 (%)  NKT IL-9 (MFI) | 0.2 (0-0.4)  739 (0-1379) | 0.09 (0.07-0.10)  758 (599-909) |
| NKT IL-10 (%)  NKT IL-10 (MFI) | 0.8 (0.6-3.5)^0.07^  6395 (5064-8809)* | 0.45 (0.40-065)  5613 (5216-5944) |
| T IFN-γ (%)  T IFN-γ (MFI) | 14.9 (9.5-19.7)  5504 (4575-6611) | 18.6 (16.3-24.2)  7736 (7397-8246) |
| T IL-9 (%)  T IL-9 (MFI) | 0.02 (0.01-0.1)  629 (551-660) | 0.02 (0.02-0.03)  692 (652-726) |
| T IL-10 (%)  T IL-10 (MFI) | 0.4 (0.3-0.9)  6881 (5068-7818) | 0.30 (0.23-0.30)  7383 (7345-7470) |

**Supplementary Table 12.** Comparison between T, NK, NKT-like, and γδ T cell populations and cytokine production at baseline, after one (T1M), and four months (T4M) of treatment with apremilast in patients with PsA.

* p < 0.05 comparing T1M and baseline; ** p < 0.05 comparing T4M and baseline; # p < 0.05 comparing responder vs non-responder at baseline; ^p < 0.05 comparing responder vs non-responder at T1M; $ p < 0.05 comparing responder vs non-responder at T4M

|  |  | **Baseline** | **T1M** | **T4M** |
| --- | --- | --- | --- | --- |
| γδ T cells | Responder (n = 17) | 0.60 (0.30-0.90) ^#^ | 0.70 (0.20-0.80) | 0.5 (0.20-0.60)^$^ |
|  | Non-responder (n = 6) | 1.2 (0.9-1.6) | 0.8 (0.6-1.8) | 1.4 (0.7-2.2) |
| NK cells | Responder (n = 17) | 7.5 (3.50-9.20) | 4.5 (2.70-7.80) | 4.8 (3.0-9.80) |
|  | Non-responder (n = 6) | 4.4 (3.4-5.7) | 4.1 (0.7-6.6) | 5.9 (4.2-21.6) |
| NKT cells | Responder (n = 17) | 1.30 (1.00-4.20) | 1.1 (0.70-2.20) | 1.1 (0.80-4.10) |
|  | Non-responder (n = 6) | 2.2 (1.5-2.9) | 1.3 (1-3.3) | 3.5 (1.7-6.4) |
| T cells | Responder (n = 17) | 76.4 (67.2-83.2) | 79.4 (74.20-87.50) | 83.3 (74.7-83.80) |
|  | Non-responder (n = 6) | 83.9 (77.2-87.9) | 87.8 (80.7-93.2) | 81.2 (63.6-89.9) |
| γδ T IFN-γ (%)  γδ T IFN-γ (MFI) | Responder (n = 17) | 36.8 (11.4-43.1)  5441 (4090-6680)^#^ | 22.1 (3.20-30.5)*  4124 (2225-6555) | 5.50 (4.50-13.80)**  1966 (1831-2114)** |
|  | Non-responder (n = 6) | 29.4 (24.5-31.6)  3164 (2654-4623) | 27.3 (24.9-30.9)  2272 (2017-6578) | 23.6 (10.1-32.8)  2068 (1989-2094) |
| γδ T IL-9 (%)  γδ T IL-9 (MFI) | Responder (n = 17) | 0.50 (0.00-1.20)  593 (0-850) | 0 (0-0.05)^  0 (0-1839) | 0 (0-0.20)  0 (0-970) |
|  | Non-responder (n = 6) | 0 (0-0.5)  0 (0-528.8) | 0.08 (0.08-0.2)  1154 (336-1636) | 0.09 (0.06-0.5)  344 (312-367.3) |
| γδ T IL-10 (%)  γδ T IL-10 (MFI) | Responder (n = 17) | 1.60 (0.75-2.30)  7042 (3345-8294) | 5.30 (1.80-6.70)  2222 (1927-2494) | 1.70 (1.20-3.10)  2773 (2095-3140) |
|  | Non-responder (n = 6) | 3.1 (0.7-14.8)  4213 (3354-4706) | 1.3 (1.3-3.8)  2746 (2297-3619) | 1.7 (1.1-4.9)  2461 (2233-2707) |
| NK IFN-γ (%)  NK IFN-γ (MFI) | Responder (n = 17) | 35.8 (22.3-42.0)  3089 (3221-2792)^#^ | 32.4 (23.6-53.5)^  3514 (3045-3538)^ | 33.9 (25.4-50.4)^$^  3507 (3189-4118)^$^ |
|  | Non-responder (n = 6) | 11.5 (7.3-22.0)  2173 (1962-2274) | 8.8 (6.6-9.5)  2129 (2049-2297) | 10 (4.3-13.5)  2146 (2052-2192) |
| NK IL-9 (%)  NK IL-9 (MFI) | Responder (n = 17) | 0.20 (0.15-0.55)  567 (542-894) | 0.20 (0.08-0.30) 542 (516-593) | 0.30 (0.10-0.40)^$^  718 (590-977)^$^ |
|  | Non-responder (n = 6) | 0.05 (0.01-0.3)  532 (119-665.3) | 0.04 (0.02-0.08) 897 (493-2544) | 0.05 (0.01-0.07)  662.5 (463.8-1536) |
| NK IL-10 (%)  NK IL-10 (MFI) | Responder (n = 17) | 0.40 (0.30-1.40)  5328 (4971-6139)^#^ | 0.50 (0.30-0.80)  3380 (3055-3824) | 0.50 (0.30-0.90)  3341 (2910-3769) |
|  | Non-responder (n = 6) | 1 (0.3-3.7)  3765 (3014-4596) | 0.4 (0.4-1.1)  3494 (3325-6535) | 0.5 (0.4-0.6)  3378 (3078-5018) |
| NKT IFN-γ (%)  NKT IFN-γ (MFI) | Responder (n = 17) | 51.2 (31.4-64.5)  5393 (5023-6444)^#^ | 61.7 (41.1-69.0)  6474 (5080-6718)^ | 49.2 (39.5-62.7)  6632 (6305-7821)^$^ |
|  | Non-responder (n = 6) | 56.3 (41.7-65.2)  4157 (3192-4585) | 35.1 (33.4-44.9)  3477 (3302-5088) | 42.5 (18.2-44.8)  3092 (2653-3341) |
| NKT IL-9 (%)  NKT IL-9 (MFI) | Responder (n = 17) | 0.20 (0.00-0.35)  736 (0-739) | 0.30 (0.06-0.30)  1767 (1023-3938) | 0.50 (0.30-1.0)  1579 (791-2387) |
|  | Non-responder (n = 6) | 0.3 (0.05-0.8)  1259 (205.3-14590) | 0.08 (0.03-0.4)  2225 (560-3751) | 0.07 (0.02-0.02)  1016 (584.3-1148) |
| NKT IL-10 (%)  NKT IL-10 (MFI) | Responder (n = 17) | 0.60 (0.40-0.75)  8180 (7197-9729)^#^ | 1.00 (0.60-1.60)  4603 (4246-5324) | 1.50 (0.60-1.90)  5341 (4867-5446)^$^ |
|  | Non-responder (n = 6) | 3.5 (1.2-12.5)  5064 (4251-5398) | 0.4 (0.4-0.6)  4690 (3847-6535) | 0.5 (0.4-0.9)  4038 (3856-5926) |
| T IFN-γ (%)  T IFN-γ (MFI) | Responder (n = 17) | 14.9 (9.3-19.3)  6351 (5540-7604)^#^ | 17.0 (12.2-22.3)  6758 (6442-8096)^ | 17.6 (15.3-22.8)^$^  7889 (7507-8948)^$^ |
|  | Non-responder (n = 6) | 14.8 (9.5-22.1)  4252 (3450-4732) | 16.9 (6.9-25.3)  4174 (3417-5001) | 9.4 (6.2-11.4)  3682 (2947-4118) |
| T IL-9 (%)  T IL-9 (MFI) | Responder (n = 17) | 0.05 (0.02-0.09)  642 (551-669) | 0.04 (0.03-0.09)  746 (609-1002) | 0.06 (0.03-0.10)  683 (635-713) |
|  | Non-responder (n = 6) | 0.009 (0.002-0.01)  592 (138.8-652.3) | 0,008 (0,007-0.02)  578 (565-615) | 0.02 (0.01-0.04)  564.5 (511.3-602) |
| T IL-10 (%)  T IL-10 (MFI) | Responder (n = 17) | 0.40 (0.20-0.40)  7337 (6881-9032) | 0.40 (0.30-0.50)  6494 (5750-6766)* | 0.50 (0.40-0.50)  7153 (6507-7677) |
|  | Non-responder (n = 6) | 1. (0.3-12.4)   4976(4864-5858) | 0.3 (0.3-0.4)  5142 (5956-5541) | 0.4 (0.3-0.5)  4869 (4409-5066) |

**Supplementary Figure 1.** Macrophage differentiation morphology observed at different stages under light microscopy 20x; Panel A. M1 macrophages differentiation; Panel B. M2 macrophage differentiation.


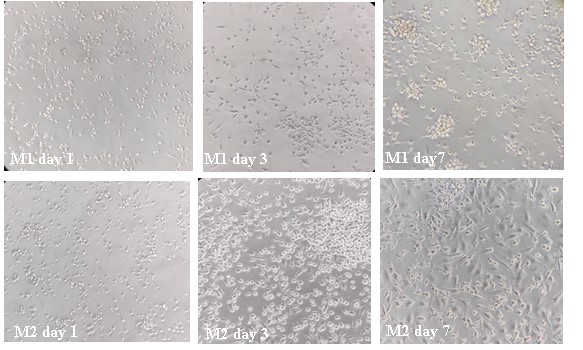


Panel A

Panel B

**Supplementary** **Figure 2.** Gene expression (reported as fold change) and supernatant levels (pg/mL for IL-10 and TGF-β, and ng/mL for IL-1Ra) of regulatory cytokines (IL-10, TGF-β, IL-1Ra) from monocyte-derived M2 macrophages. Data are reported for OA controls and patients with PsA at the baseline condition (T0), after 7 days (T7D), one month (T1M) and 4 months (T4M) of treatment with apremilast. * p < 0.05 comparing T0 and T4M in patients with PsA

**
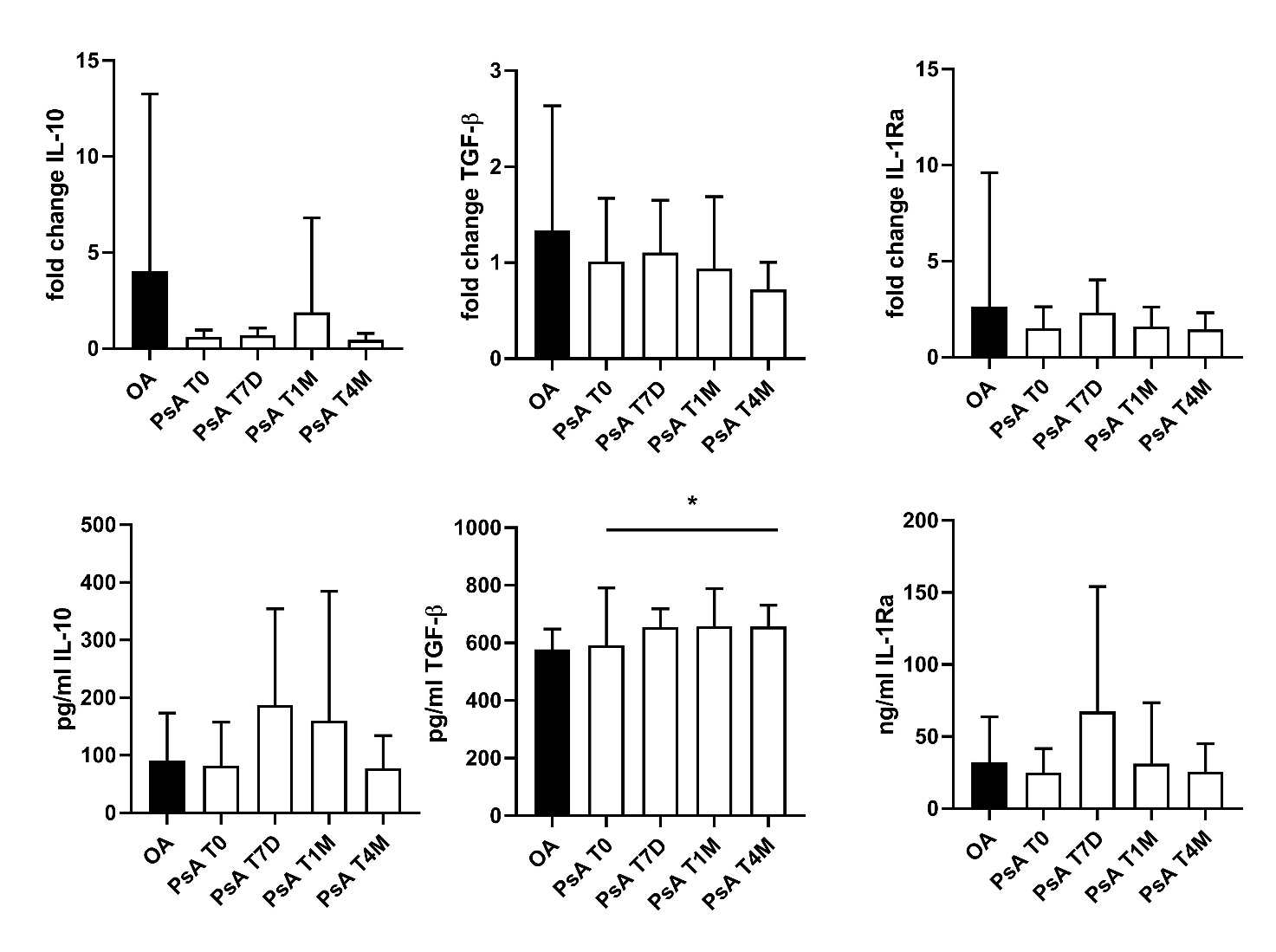
**

**Supplementary** **Figure 3.** Gene expression (reported as fold change) and supernatant levels (pg/mL for IL-10 and TGF-β, ng/mL for IL-1Ra) of regulatory cytokines (IL-10, TGF-β, and IL-1Ra) from monocyte-derived M2 macrophages in patients responding and not responding to apremilast. Data are reported for the baseline condition (T0), after 7 days (T7D), one month (T1M) and 4 months (T4M) of treatment with apremilast.


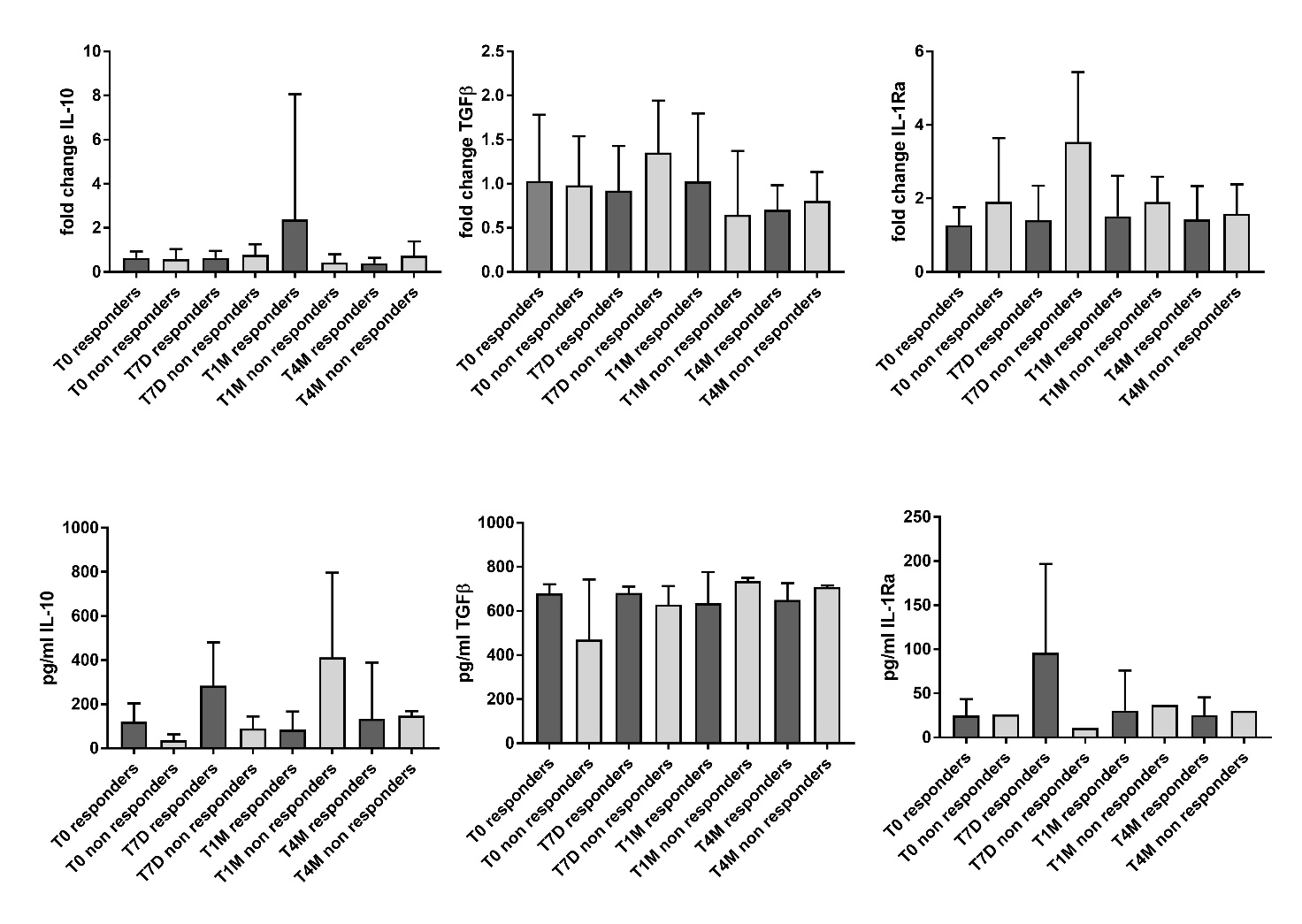

Supplement: Supplementary file 1 [file DataSheet1.docx]
